# Plant-based solutions for veterinary immunotherapeutics and prophylactics

**DOI:** 10.1186/s13567-014-0117-4

**Published:** 2014-12-31

**Authors:** Igor Kolotilin, Ed Topp, Eric Cox, Bert Devriendt, Udo Conrad, Jussi Joensuu, Eva Stöger, Heribert Warzecha, Tim McAllister, Andrew Potter, Michael D McLean, J Christopher Hall, Rima Menassa

**Affiliations:** Department of Biology, University of Western Ontario, 1151 Richmond St, London, ON Canada; AAFC, Southern Crop Protection and Food Research Centre, 1391 Sandford St, London, ON Canada; Laboratory of Immunology, Faculty of Veterinary Medicine, Ghent University, Salisburylaan 133, 9820 Merelbeke, Belgium; Leibniz Institute of Plant Genetics and Crop Plant Research, Gatersleben, Germany; VTT Technical Research Centre of Finland, Espoo, Finland; Department for Applied Genetics and Cell Biology, University of Natural Resources and Life Sciences, Vienna, Austria; Technische Universität Darmstadt, FB Biologie, Schnittspahnstr. 5, D-64287 Darmstadt, Germany; AAFC, Lethbridge Research Centre, 5403, 1 Avenue South, Lethbridge, Alberta Canada; Vaccine and Infectious Disease Organization (VIDO), University of Saskatchewan, 120 Veterinary Road, Saskatoon, Saskatchewan Canada; Department of Veterinary Microbiology, University of Saskatchewan, 120 Veterinary Road, Saskatoon, Saskatchewan Canada; PlantForm Corp., c/o Room 2218, E.C. Bovey Bldg, University of Guelph, Guelph, Ontario N1G 2 W1 Canada; School of Environmental Sciences, University of Guelph, 50 Stone Road East, Guelph, Ontario N1G 2 W1 Canada

## Abstract

An alarming increase in emergence of antibiotic resistance among pathogens worldwide has become a serious threat to our ability to treat infectious diseases according to the World Health Organization. Extensive use of antibiotics by livestock producers promotes the spread of new resistant strains, some of zoonotic concern, which increases food-borne illness in humans and causes significant economic burden on healthcare systems. Furthermore, consumer preferences for meat/poultry/fish produced without the use of antibiotics shape today’s market demand. So, it is viewed as inevitable by the One Health Initiative that humans need to reduce the use of antibiotics and turn to alternative, improved means to control disease: vaccination and prophylactics. Besides the intense research focused on novel therapeutic molecules, both these strategies rely heavily on the availability of cost-effective, efficient and scalable production platforms which will allow large-volume manufacturing for vaccines, antibodies and other biopharmaceuticals. Within this context, plant-based platforms for production of recombinant therapeutic proteins offer significant advantages over conventional expression systems, including lack of animal pathogens, low production costs, fast turnaround and response times and rapid, nearly-unlimited scalability. Also, because dried leaves and seeds can be stored at room temperature for lengthy periods without loss of recombinant proteins, plant expression systems have the potential to offer lucrative benefits from the development of edible vaccines and prophylactics, as these would not require “cold chain” storage and transportation, and could be administered in mass volumes with minimal processing. Several biotechnology companies currently have developed and adopted plant-based platforms for commercial production of recombinant protein therapeutics. In this manuscript, we outline the challenges in the process of livestock immunization as well as the current plant biotechnology developments aimed to address these challenges.

## Table of contents

1. Introduction
2. Immunization of livestock animals
  2.1 Active/passive immunization
  2.2 Induction of protective immunity
  2.3 Modes of vaccination
    2.3.1 Subcutaneous and intramuscular
    2.3.2 Intranasal
    2.3.3 Oral
3. Plant-based bioreactors
4. Post-translational protein modifications in plants
5. Opportunities and advantages of plant systems
  5.1 Storage/Shelf life/Purification
  5.2 Glycoengineering
  5.3 Vaccine bioencapsulation and delivery
  5.4 Scale-up and speed
6. Examples of therapeutic proteins produced in plants
  6.1 Antibodies
  6.2 Antigens: VLPs
  6.3 Subunit vaccines
    6.3.1 Poultry
    6.3.2 Swine
    6.3.3 Cattle
  6.4 Toxic proteins
7. Conclusions
8. List of abbreviations
9. Competing interests
10. Authors’ contributions
11. Acknowledgements
12. References

## 1. Introduction

The health and well-being of food-bearing animals is a major preoccupation for any livestock, poultry or fish producer. Endemic disease or epidemic outbreaks represent a very significant financial risk to the producer due to loss of animals, production of animals that are not marketable, and reduction in feed conversion efficiency. The potential risk to consumers from contaminated food is an important public health concern, and very significant investments are made by the agri-food industry to ensure safe food products. Nevertheless, an estimated 9.4 million cases of illness due to consumption of food contaminated with known pathogens occurs annually in the United States [1]. Thus, it is critically important that primary producers ensure the health of their livestock for public health, animal welfare and business profitability reasons.

The key to minimizing animal morbidity and mortality is the employment of good production practices. Best practices will vary according to the production system, but land-based agriculture will typically include provision of uncontaminated feed and water, adequate ventilation and air quality, biosecurity, robust surveillance of animal health, and the judicious use of antimicrobial agents and parasiticides for disease prevention and treatment, when warranted. Prominent in the animal health toolbox are antibiotics. It can be reasonable to assume that the availability of antibiotics will become increasingly constrained as legitimate public alarm over the looming spectre of catastrophic antibiotic resistance in human medicine is translated into action at the farm level. Furthermore, the development of antibiotic resistance will progressively reduce antibiotic therapy options available to veterinarians. More restricted use of veterinary antibiotics will result from market-driven forces, as consumers increasingly demand antibiotic-free food, and through the policies of governments and codes of practice of veterinary practitioners that promote judicious use [2]. Within this evolving environment, newly developed vaccines and immunotherapeutic agents offer the potential to lessen the need for antibiotics for disease control, and offer veterinary practitioners much needed tools [3].

The global market for animal vaccines is estimated to be currently worth $5.8 billion, and is anticipated to grow with a compound annual growth rate of 8.1% to a value of $8.6 billion by 2018 [4]. The efficacy of a vaccine, the ease with which it can be employed, and the overall benefit in terms of increased productivity must be competitive with other disease management options. Recent advances in immunology and in biotechnology, specifically the development of methods to produce potent vaccines very cost-effectively using plant-based bioreactors have the potential to make vaccines an even more attractive proposition. Furthermore, in cases where vaccination cannot be used to prevent disease, the production of antibodies in plants for passive immunotherapy and infectious disease control holds great promise.

This review paper will cover animal immunization essentials and will present the latest developments in plant biotechnology for the production of veterinary therapeutics. Specific aspects discussed will include high-level recombinant protein production in plant-based systems, the ability to use unpurified material for treatment, the possibility of oral rather than parenteral delivery, low cost of protein/vaccine production, very short turnaround time from conception to large-scale manufacturing, and the ability to “stack” genes encoding antigens and adjuvants to create potent multi-target vaccines in a single preparation.

## 2. Immunization of livestock animals

### 2.1 Active/passive immunization

In contrast to humans, newborn farm animals (foals, piglets, calves, lambs, kids) depend totally, and companion animals (kittens, puppies) almost completely upon uptake of colostral antibodies for their systemic maternal immunity against pathogens [5]. Intestinal uptake occurs via endocytosis, involving the neonatal Fc receptor (FcRn) and is highest immediately after birth and declines rapidly within 24 h as intestinal cells mature and intestinal flora are established. The time of cessation of this macromolecular transport (gut closure) varies by species and immunoglobulin type, ranging between 20 to 36 h of age [6]. For multiparous animal species, the degree of passive immunity can be heterogeneous between litters and among members of a same litter because of variability in colostral absorption. This is true for piglets, puppies and kittens, whose levels of passive antibodies are maximum 36-48 h after birth. From this time on, the immunoglobulin concentrations decline in the serum of the newborn animal. The half-life depends on immunoglobulin isotype, and on the species, varying between 1 to 4 days for IgA and IgM, to approximately 8 to 22 days for canine and equine IgG, respectively [7]. The affinity of IgG for endothelial FcRn most likely accounts for these differences observed in serum half-life among species and different IgG subclasses [7,8]. Also, the growth rate of the animal influences the rate of decline in the level of the maternally-derived antibodies. This is particularly clear in canine species, where dogs belonging to rapid growth breeds eliminate their maternally-derived antibodies more quickly than slow-growth breeds. In addition to differences in uptake of maternal antibodies, the degree of passive immunity is not equal for all tissues. For instance, colostral immunity protects young animals against systemic infection during the first months of life, whereas the respiratory tract mucus is protected only for a few weeks [9].

Shortly after parturition, drastic changes occur in the composition of colostrum with a drastic drop in IgG and IgA. In most domestic animals, the drop in IgA is less pronounced than IgG, so that IgA becomes the most important immunoglobulin in milk [10]. Milk antibodies are essential to protect young animals against intestinal infections. The IgA in the milk is dimeric secretory IgA (SIgA). The dimeric IgA is secreted in the mammary gland tissue by plasma cells, binds with its J chain to the polymeric immunoglobulin receptor (pIgR) expressed at the basolateral site of the alveolar epithelial cells and is endocytosed, transcytosed and exocytosed to the interstitial space after cleavage of the receptor. The extracellular 80 kDa part of pIgR is exceptionally carbohydrate-rich, and when incorporated into the Ig molecules as bound secretory component (SC), it endows SIgA with resistance against proteolytic degradation [11]. In ruminants, IgG concentrations remain higher than IgA, and IgG1 is specifically enriched in colostrum and milk, with levels approximately 10 times higher than IgA or IgG2. The role of FcRn in this transport has not been completely clarified [7,8].

### 2.2 Induction of protective immunity

At weaning, lactogenic protection disappears, making the young animals highly susceptible to infections with enteropathogens. Most pathogens either colonize the mucosa or invade the host at a mucosal surface. Optimal protection against these pathogens most often involves the induction of pathogen-specific SIgA at the infection site [12]. As such, an optimal vaccine should induce robust mucosal SIgA immune responses.

Parenteral vaccines typically induce IgG, a response that is not particularly effective against these types of infections. The best way to elicit pathogen-specific SIgA is to activate the local mucosa-associated lymphoid tissue (MALT) at the site of colonization or invasion through mucosal vaccination. This necessity to administer vaccines at the mucosal site relating to the pathogen’s tropism is further highlighted by the compartmentalization of the mucosal immune system. For example, stimulation of gut-associated lymphoid tissue (GALT) does not induce a protective SIgA response in the respiratory tract, while induction of an immune response at the nasal mucosa fails to induce intestinal protection [11,13]. Taking this into account, the induction of mucosal immunity further requires the vaccine to pass several physiological barriers and defence mechanisms. To reach the inductive sites of the mucosal immune system, whether it is upon oral, intranasal, rectal or urogenital administration, the vaccine has to surmount mucus and antimicrobial peptides and traverse the epithelial barrier. In case of oral administration, the vaccine also needs to survive the low pH in the stomach, digestive enzymes and bile salts and be transported via peristalsis to the GALT. Furthermore, the vaccine has to trigger protective mucosal immunity instead of tolerance, which is the dominant response mode of the mucosal immune system, and this without causing severe inflammation [13]. Fusing vaccine antigens to antibody or antibody fragment to target the subunit vaccine to antigen-sampling routes at the mucosal surfaces, such as transcytotic epithelial receptors, could drastically improve oral vaccine efficacy [14].

Only a limited number of mucosal vaccines have been shown to meet these criteria and, consequently, only a few have been internationally licensed. Of those that have been licensed, most are based on live-attenuated pathogens and a few are inactivated pathogens [15]. No mucosal subunit vaccines have been licensed so far. Whereas many particle systems, such as virus-like particles (VLPs, see text) or other particles have been shown to be immunogenic in mice via the oral route, almost none of these were successful in larger animals or humans [3]. This could be explained by the longer intestinal transit time for these particles to reach the inductive sites in larger mammalian species in contrast with mice. As a result, in larger animals, only a small portion of the particles will reach the GALT, resulting in fewer particles being taken up by M cells and subsequently no or low induction of intestinal mucosal immune responses.

In contrast to particulate systems, soluble antigens are not immunogenic and in general induce tolerance upon mucosal administration even in mice. However, a few soluble antigens have been shown to be immunogenic via the mucosal route, such as cholera toxin (CT), thermolabile enterotoxin (LT) and their B subunits (CTB and LTB) [16], and the purified F4 fimbriae of enterotoxigenic *E. coli* in pigs [17]. These antigens are characterized by the ability to bind to receptors on enterocytes in the small intestine and to be taken up by these epithelial cells [18], followed by induction of an antigen-specific IgA response. Whereas microgram quantities of CTB and LTB are immunogenic, F4 has to be given in milligram amounts [17]. The oral route has the advantage over other routes in that the vaccine antigens can be administered in the feed, eliminating the need to restrain livestock during administration and allowing the use of less-purified antigens.

### 2.3 Modes of vaccination

#### 2.3.1 Subcutaneous and intramuscular

The delivery of veterinary vaccines, in general, is by injection although there are variations depending upon the animal species being immunized, management practices and age of the animal. For example, virtually all vaccines for cattle are delivered parenterally, while those for use in poultry are delivered by injection, orally, or by aerosol. Ultimately, the route of delivery is dictated largely by the various management systems employed, as well as the ease of handling of individual animals.

There are a number of issues associated with invasive vaccine delivery in food-producing animals, one of which is the economic loss associated with trimming of area surrounding injection site lesions. Many veterinary vaccines are extremely pro-inflammatory in nature, an example being Clostridial vaccines used in cattle. When delivered subcutaneously, these vaccines tend to result in a large swelling which can persist for months. On the other hand, intramuscular injection does not result in overt swelling but causes significant tissue damage near the injection site. The cost associated with carcass trimming has been estimated to be between $1.46 and $40.00 per animal [19]. In addition, it is not uncommon for needles to break during intramuscular delivery, causing problems at processing, as well as the retail level. While injection of the antigens themselves can result in tissue damage, most killed vaccines also contain strong adjuvants which contribute to this damage. Adjuvants significantly increase the cost of the vaccines and also dictate both the quality and magnitude of the immune response, the former of which has been biased towards Th2 immune responses. Adjuvants currently in use are suitable for injection, but most are not compatible with mucosal delivery [20]. To maintain their efficacy, most injectable vaccines require proper refrigeration and storage, a need that can be challenging in extensive livestock operations especially in developing nations.

#### 2.3.2 Intranasal

The delivery of modified live viral vaccines via the intranasal route or in drinking water has been effective. However, live bacterial vaccines are not commonly used, in part due to the use of antibiotics in the industry. Mucosal vaccines are available for swine, including those for *Bordetella bronchiseptica*, where they have been shown to result in enhanced SIgA production following a single administration by the intranasal route [21]. A novel adjuvant platform consisting of CpG ODNs, polyphosphazenes and cationic innate defense regulator peptide (IDR) 1002, has also been tested for efficacy in a swine model and shown to provide a long duration of immunity against *Bordetella pertussis* in mice following a single intranasal administration [22]. Interestingly, this formulation also works in the presence of maternal antibodies [23], making it ideal for mucosal immunization of neonates.

All presently available commercial vaccines targeting the respiratory tract via intranasal, aerosol or conjunctival application are live attenuated or vector vaccines [24]. Nevertheless, there is the potential for using protein vaccines consisting of dead microorganisms or subunits (virulence factors). Immunization of the respiratory tract has been experimentally demonstrated by i) using particulate delivery systems, which are either lipophilic, mucoadhesive and/or contain immunomodulating molecules, ii) using soluble conjugates of antigens (or epitopes) and a carrier, targeting the antigens to the mucosa of the respiratory tract or iii) using soluble antigens. Often, adjuvants are needed for strong mucosal immune responses [25]. Whereas experimental results have been promising in many studies, no dead vaccines for immunization of the respiratory tract have been commercialized, as adverse reactions have hampered their development. Indeed, anaphylaxis due to impurities, or Bell’s palsy due to vaccine components have been described [26]. Results from animal studies suggest that the intranasal route may itself facilitate transport of proteins, inactivated vaccines, live viruses or other particles into the central nervous system [27]. Therefore, candidate vaccines targeting the respiratory tract mucosa have to be thoroughly evaluated for adverse effects.

#### 2.3.3 Oral

Oral vaccination, or so called “edible vaccines”, have been traditionally viewed as the panacea of plant-produced vaccines in terms of their use as an approach to disease control [28]. Edible vaccines offer several advantages over conventional methods of vaccine delivery. Edible vaccines come in contact with the lining of the digestive tract, thus giving them the potential to stimulate both mucosal and systemic immunity, the former of which is a response that is notoriously difficult to achieve through injections, which bypass mucosal barriers. In part, this accounts for the reason as to why the efficacy of injectable vaccines against intestinal pathogens has generally been quite poor.

Edible vaccines also have positive implications for livestock welfare, as they could be administered directly in the diet, eliminating the need to confine the animal, or the need to breach the skin through injection, a practice that can promote secondary infections. Oral administration of vaccines eliminates the risks that injection-based methods pose on meat quality as a result of intramuscular administration.

## 3. Plant-based bioreactors

Being eukaryotic organisms, plants can express, synthesize and process complex heterologous proteins similarly to “conventional”, fermentation-based expression systems, such as mammalian, insect and yeast cell cultures. Yet, plants have some variances in the pattern of protein glycosylation [29]. Production of recombinant therapeutic proteins has been demonstrated in a variety of plant species. Plant cells contain genomes of three types: nuclear, chloroplast (plastome) and mitochondrial genomes. Generation of stable transgenic plants that produce recombinant proteins can be efficiently achieved through genetic engineering of the nuclear genome or the plastome. In addition, transient expression methods allow quick and robust production of recombinant proteins by way of infiltration of plant leaf tissue with transgene-harboring agrobacteria [30].

Recombinant proteins produced in nuclear-transformed plants are synthesized in the cytoplasm and can be secreted, or accumulated in different sub-cellular organelles with the use of appropriate transit or signal peptides. Using tissue-specific promoters enables spatially-defined expression of the transgene in applicable plant organs, such as leaves, seeds, fruits or tubers [31]. Nuclear-transformed plants offer tremendous simplicity for production of a vaccine product, as seed lines can be developed and propagation of plant biomass can utilize traditional agricultural technologies and infrastructure. Note, however, that nuclear-transformed plants typically express lower yields of recombinant proteins compared with transient expression, require prolonged timeframes for generation and selection of transgenic seed lines, and require measures to prevent environmental contamination due to transgene flow via open field cross-pollination [32].

Transgenic plants with transformed plastome (transplastomic plants) possess several beneficial traits, such as i) high-ploidy of plastome and lack of positional effects or transgene silencing, leading to synthesis of typically very high levels of recombinant proteins, ii) the ability to express artificial operons, thus allowing expression of several proteins in one transformational step, and iii) tight transgene containment due to maternal inheritance of plastid DNA in most cultivated plants [33]. The absence of a glycosylation machinery in chloroplasts thwarts the expression of proteins where glycans are essential for structure/conformation; however, this technology eliminates risks of addition of potentially allergenic non-mammalian glycans and offers advantages for expression of non-glycosylated proteins of prokaryotic origin [34].

High yields of recombinant proteins can be obtained quickly with transient expression systems through use of viral, virus-derived and non-viral expression vectors [30]. Infiltration of the leaf tissue with an *Agrobacterium* intermediate host containing any of these types of expression vectors (agroinfiltration) is the most common method used for delivery of foreign DNA into plant cells. The main advantage of the transient expression systems is the production speed that enables vaccines to be manufactured within periods as short as one month after obtaining the sequence of the antigen [35]. However, transient expression systems are labor- and material-intensive procedures involving growing *Agrobacterium* cultures, and diluting them in infiltration cocktails, immersion of plant aerial biomass into the Agrobacterium suspension, infiltrating the agrobacteria into leaves by pulling and releasing a vacuum to allow entry of *Agrobacterium* into the plant tissues [36]. These manipulations add cost to recombinant protein production, but have the advantage of using wild type plants, which is viewed by regulatory authorities favorably. The US Defense Advanced Research Program Agency (DARPA) has funded major infrastructure projects, such as Medicago’s [37] commercial-scale production facility in the Research Triangle Park, NC, and Kentucky BioProcessing’s [38] production facility in Owensboro, KY. Also, an improved viral vector-based inducible system that can be deployed for field production has recently been developed [39].

Other plant-based production systems include algae and moss cell-suspension cultures grown in bioreactors. High-level containment and sterile growth conditions are advantages of these systems, compared to whole plants in terms of good manufacturing practice (GMP) for recombinant protein production, though the cost of these systems is high, while scale-up ability is limited [40].

## 4. Post-translational protein modifications in plants

Although the genetic code is universal and a given nucleotide sequence results in the same order of amino acids in the polypeptide regardless of what organism is translating the RNA message, proteins differ in structure depending on the organism they are produced in. This is due to the fact that a multitude of modifications can take place after the ribosomal synthesis of the polypeptide, so called post-translational modifications (PTMs). In the case of therapeutic proteins these PTMs are of high importance, since they might alter the structure of the protein, its functionality and especially its immunogenicity [41]. PTMs include glycosylation, phosphorylation, lipidation, disulfide-bridging (to name only the most prominent), which basically occur in vivo, but some might also occur during downstream processing of a given therapeutic protein. Different organisms are capable of various PTMs, e.g. bacteria do not glycosylate proteins, while eukaryotic organisms perform extensive glycomodifications. Moreover, type and linkage of sugars vary considerably among cell types and organs, as well as at different developmental stages and environmental conditions, making this particular modification highly diverse. In the case of recombinant therapeutics for parenteral administration, it is of importance to retain the native glycosylation pattern to avoid shortened half-life and unintended immunogenicity. Plants do glycosylate recombinant proteins in a manner that differs from that in mammalian cell systems, and glycosylation may even vary among plant species, organs and cell types. In some cases this can even be an advantage, exemplified by a recombinant glucocerebrosidase (taliglucerase alfa) made in carrot cells. Here, the sugars added to the produced recombinant enzyme more closely resemble the native structure with terminal mannose, making this product superior or “biobetter”, compared to the CHO cells-derived product [42]. As a further improvement, glycosylation machinery in plants can be engineered to produce mammalian-like glycan patterns with terminal sialic acid, making the recombinant proteins almost indistinguishable from those produced in mammalian cell lines (see “Glycoengineering” in text).

## 5. Opportunities and advantages of plant systems

### 5.1 Storage/Shelf life/Purification

Plant-based expression systems raise the possibility that antigens or antibodies can be produced in a form that is stable during storage and is amenable to extraction and purification procedures [43]. Dried or lyophilized leafy biomass, as well as plant storage tissues, such as seeds, retain unchanged levels of accumulated recombinant proteins for years at normal room temperatures, thus reducing stowing costs and facilitating distribution without need for a cold chain [44,45]. Such a production system would allow stockpiling of the transgenic plant material (lyophilized leaf biomass or seeds) for manufacturing of rapid-response veterinary biologics targeted against pathogens of epidemiological relevance. The time to product would then only be dependent on the speed of extraction and downstream processing, if purification is required, or immediate, if formulated lyophilized biomass or whole seeds can be administered in feed [46]. Some plant-based expression strategies, such as the expression of recombinant antigens in oil bodies or implementation of particular protein tags are specifically focused at overcoming the complexities of purification procedures (reviewed in [47]). Although purified plant-derived biologics are functional and effective, a key benefit of plants is the ability to deliver recombinant pharmaceuticals as minimally processed feed, such as flour paste from edible tissues [48]. Expression in seeds may also help protect antigens from digestion within the intestinal tract, especially if they target the formation of protein bodies, which can increase the delivery of antigens to the mucosal surface [49]. Remarkably, the formation of ectopic storage organelles as sink compartments for recombinant proteins can be induced in tissues that are not adapted for storage functions [50].

### 5.2 Glycoengineering

Glycosylation affects the quality of recombinant pharmaceuticals as different glycan structures can potentially influence stability, subcellular targeting, immunogenicity, pharmacokinetics and biological activity. The precise control and optimization of glycan modification to manufacture homogeneous protein therapeutics with specific designer glycoforms that confer superior efficacy has therefore become an important issue for all production platforms, including mammalian cells.

The mechanisms of N-glycosylation in plants and mammals differ in Golgi-specific modifications, such as the addition of β(1,2)-linked xylose and core α(1,3)-linked fucose residues in plants, and the addition of β(1,4)-linked galactose and sialic acid residues in mammals [51]. Therefore, strategies to control the glycosylation of recombinant proteins were established in plants early on, such as subcellular targeting to prevent the addition of certain sugar residues [52] and glycoengineering to replace sugars with more desirable counterparts [53]. A key example of glycan control by targeting is the first commercially available plant-derived human therapeutic protein, taliglucerase alfa, indicated for the lysosomal storage disorder called Gaucher’s disease. In this case targeting to the plant vacuole resulted in the formation of glycan structures with terminal mannose residues promoting uptake of the recombinant protein via mannose-specific surface receptors on macrophages [54].

Engineering the plant N-glycosylation pathway has been achieved by various approaches including conventional mutagenesis or homologous recombination, RNA interference and transgene expression [53]. The versatility of plants for the production of specific N-glycoforms has turned a perceived disadvantage into one of the major strengths of plant production platforms. In addition to the abolition of β(1,2)-linked xylose and core α(1,3)-linked fucose residues, and the complete reconstruction of mammalian glycosylation pathways in plants [55], product-specific designer N-glycans have been engineered for improved efficacy. For example, glycoengineered plants can be used to produce glyco-optimized antibody versions with improved pharmacokinetic properties due to greater receptor affinity [56].

Another type of glycosylation whose impact is less widely known is the mucin-like O-glycosylation of serine, threonine and hydroxyproline residues, the latter being unique to plants. However, recent efforts have addressed the engineering of O-linked glycans [57]. It has been suggested that O-glycan structures may function as adjuvants, or may increase serum stability [51]. Tailored mammal-like O-glycan structures could also improve the stability and activity of proteins such as erythropoietin and secretory IgA, giving plants a competitive edge for the production of these compounds.

### 5.3 Vaccine bioencapsulation and delivery

Although plants have been shown to manufacture biologically-active therapeutics in amounts sufficient for oral administration to livestock, plant-derived antigens require a formulation to protect them from the hostile environment of the gastro-intestinal tract, without interfering with the immunogenicity of the antigen. Depending on the plant species and the plant tissue in which the subunit proteins are expressed, the plant matrix could provide some protection against these harsh conditions. Not only does this eliminate the need for costly purification, but the provision of a durable matrix offers a protective and stabilizing effect beyond harvest and upon mucosal administration [58]. Any plant cell matrix can be effective to enhance resistance against digestion, thereby increasing exposure of a vaccine to immune effector cells, but the protective effect can be further enhanced by incorporation of the vaccine proteins into storage organelles [59]. The use of seeds is particularly suitable to achieve bioencapsulation of recombinant proteins in specialized storage organelles that are derived from the endomembrane system and allow proteins to accumulate within a protective matrix [52]. Clear benefits have been demonstrated in seed-based production systems as recombinant proteins expressed in rice or pea seeds were better protected from degradation than their purified counterparts upon oral delivery [60,61]. Nevertheless, plant-based production could also negatively influence the immunogenicity of the antigen by influencing antigen folding, glycosylation and/or by interaction with the antigen, interfering with its capacity to target the mucosa [46]. The latter could be one of the reasons why F4 fimbriae to which alfalfa crushed leaf biomass was added, showed a decreased immune response in pigs in comparison with F4 without this plant material (Cox et al., unpublished results).

### 5.4 Scale-up and speed

Scaling up production of recombinant proteins in transgenic or transplastomic plants is much easier and less costly than similar scaling up in mammalian or microbial cells for the simple reason that each plant could be considered as a bioreactor and all that is required is sowing a seed and providing light, water and fertilizer, as opposed to an expensive upfront investment in infrastructure and bioreactor facilities for cell cultures and the formulation of complex cell culture media. Therefore, scaling up or down can be achieved quickly with no major investment. A single tobacco plant can produce over 150 000 seeds, allowing fast scale-up within a few months. Tobacco biomass yields can reach up to 100 tons/ha in a single field season [62]. If containment is necessary, a similar or higher yield is achievable in greenhouses; and high level accumulation of the desired protein at 1 g/kg fresh leaf weight means that 100 kg of a protein can be produced in a single hectare (Menassa et al., unpublished data). However, using stable transgenic/transplastomic lines implies a lag time in producing a high expressing line and obtaining seed. In the case of a catastrophic epidemic or pandemic, much faster timelines are required. For this, transient production in *Nicotiana benthamiana* is ideal with a timeline from cloning of the genes to vaccine production of only a few weeks [35]. As well, this system obviates the need for production of transgenic plants, thus easing the regulatory framework for scale up. In this system, wild type *Nicotiana benthamiana* plants are grown in greenhouses and infiltrated with *Agrobacterium* cultures containing the expression construct required for recombinant protein production. The infiltrated plants are harvested 4-10 days post-infiltration, and the protein is purified. Several companies (Medicago Inc., Fraunhofer USA, Kentucky BioProcessing LLC) have adopted this system for production of influenza vaccines and monoclonal antibodies [35,63,64]. The MagnICON® system developed by ICON Genetics GmbH [65] allows the production and purification of monoclonal antibodies at an expected yield of 250 mg/kg plant fresh weight [63], and Medicago Inc. was able to produce 10 million doses of influenza vaccine within one month in their 97 000 square foot facility in North Carolina.

## 6. Examples of therapeutic proteins produced in plants

### 6.1 Antibodies

It has been more than 25 years since the first IgG antibody was expressed in plants. Since then, there have been numerous publications in the scientific literature on the production of antibodies in plants, such as those targeting bacteria, cancer targets and highly pathogenic viruses such as HIV and Ebola [66]. The current epidemic caused by the Ebola virus in West Africa has brought attention to a plant-produced antibody cocktail. This cocktail, called ZMAPP, was recently shown to reverse advanced Ebola disease in 100% of tested Rhesus macaques [67]. Six publications describe the production of five of 38 approved therapeutic antibodies in plants (Table 1).

**Table 1:** **PubMed listings for therapeutic antibodies produced in plants**

**Antibody (generic name)**	**Antibody (commercial name)**	**Plant species**	**Reference**
Cetuximab	Erbitux	*Zea mays*	[68]
Nimotuzumab	BIOMAb EGFR, TheraCIM, Theraloc, CIMAher	*Nicotiana tabacum*	[69]
Palivizumab	Synagis	*Nicotiana benthamiana*	[70]
Rituximab	Rituxan, Mabthera	*Lemna minor*	[56]
Trastuzumab	Herceptin	*Nicotiana benthamiana*	[71]
ZMAPP	-	*Nicotiana benthamiana*	[67]

Therapeutic and prophylactic antibodies are required in massive amounts and on a constant basis, and might therefore be produced more economically by using transgenic plants rather than fermentation. In addition, neutralizing antibodies indicated as topical or oral microbicides can also be delivered in plant material such as seeds, as demonstrated by the administration of peas containing single-chain antibodies with high sporozoite-neutralizing activity against the coccidiosis parasite, *Eimeria tenella,* for the prevention of gastrointestinal infections in chickens [60]. In another, more recent study the protection of weaned piglets against enterotoxigenic *E. coli* infection was achieved by the oral administration of seeds producing IgA [72].

Whereas simple proteins such as single chain antibody fragments tend to be easily expressed in bacteria, complex glycoproteins such as monoclonal antibodies usually require eukaryotic production systems. Full size antibodies tend to be expressed well in plants because the secretory pathway in plant cells contains an extensive molecular chaperone system. Plants are particularly beneficial for the production of secretory IgA molecules because they can produce all four of the polypeptide components and assemble a fully functional molecule [73]. In contrast, three of the protein components are produced by plasma cells in mammals, but the fourth is produced by epithelial cells, and assembly can only occur in the extracellular environment. Secretory IgA assembly can also be achieved in CHO cells carrying the four different transgenes, but the efficiency of assembly is low [74]. Therefore, plants offer a valuable and superior large-scale production system for this preferred format of mucosal antibodies.

### 6.2 Antigens: VLPs

Viral diseases are probably the most important culprit for the severe economic losses in livestock production worldwide. Although antibiotics are not used to treat or prevent viral infection outbreaks, pathogenic viruses often predispose infected animals for secondary bacterial infections, which do require antibiotic treatment. Thus, preventive vaccinations to control primary viral infections help to reduce the need for antibiotics application in livestock production.

Virus-like particles (VLPs) are defined as multisubunit protein structures with self-assembling competence that show an identical or highly similar overall structure to the corresponding native viruses [75]. Studies document a high immunogenic capacity of VLPs due to their display of multiple repeated antigenic motifs, which trigger B cell activation and elicit higher antibody titers compared to monomeric antigens. Strong humoral immune responses could be induced by VLPs without adjuvants, while avoiding stimulation of inflammatory T cell responses [76]. Many different VLPs (more than 100) originating from phages and plant, insect, and vertebrate viruses have been described and summarized in an excellent review [75], including examples of the preparative and large-scale manufacturing of VLPs in bacteria, yeast cells, insect cells and mammalian cells.

Mason et al. first demonstrated that the expression of human hepatitis B virus structural proteins in plants results in enveloped VLPs that “bud-out” as spherical particles of 22 nm in diameter off the intracellular membranes [77]. The further engineering of enveloped hepatitis B virus VLPs into nanoparticles allowed the display of large foreign antigens or the development of bivalent vaccines [78]. Simultaneous transient expression of four distinct structural proteins of Bluetongue virus (BTV) led to assembly of heteromultimeric VLPs able to elicit a strong antibody response in sheep, which provided protective immunity against BTV challenge [79]. Another example of VLPs produced in plants with improved transient expression is Norwalk virus-derived non-enveloped VLPs [80]. Lettuce containing low levels of secondary metabolites has been used to develop a robust plant virus-based production platform for VLPs derived from the Norwalk virus capsid and combined with a scalable processing method [81].

The concept of producing VLPs in plants is generally connected to the idea of edible vaccines [75]. However, there are several hurdles to resolve before this concept could be implemented, despite all of its obvious benefits. Quality control issues and precise formulation of vaccine products, as well as documented control by the relevant regulatory bodies would preclude easy on-site production in food/feed plant organs. The administration of such vaccines has to be regulated to avoid insufficient antigen application or, vice versa, to avoid the development of immune tolerance. Even orally administered VLP-type vaccines will have to be at least processed, enriched, formulated and applied under supervision, to ensure reproducible results [29].

Both the most recent and the most successful application of the plant-based VLP concept has been by Medicago Inc. (for review see [35]). A proprietary, plant-based transient expression system has been developed to produce vaccines made of budded influenza hemagglutinin particles [82]. The developed platform allowed the vaccine to be produced within one month of the disclosure of hemagglutinin sequences, from identified strains as H1N1 [82]. Because many influenza types (i.e. H5N1, H1N1) are zoonotic diseases, vaccination with plant-based VLPs has to be further developed especially in terms of lowering down-stream processing costs to fit into the economic constraints associated with a veterinary vaccine. An excellent comparative summary of different plant-based vaccines including expression levels, immunogenicity, VLP strategies and anticipated cost is given in a review by Scotti and Rybicki [83]. The authors conclude that proof of efficacy was demonstrated for several plant-based VLP vaccines and plant-based expression systems could do as well as conventional production systems based on fermentation. They claim a rather optimistic future of plant-based VLPs, if real costs are in the predicted range.

### 6.3 Subunit vaccines

The concept of using plants as expression hosts for veterinary subunit vaccines has been studied extensively within the past two decades. However, only a few studies have tested the efficacy of plant-made subunit vaccines in farm animals. This section summarizes examples showing immunization data with host species.

#### 6.3.1 Poultry

In 2006 Dow Agro Sciences obtained USDA approval for a plant cell culture-based vaccine for poultry against Newcastle disease virus [84]. This vaccine was composed of recombinant hemagglutinin-neuraminidase protein expressed in transgenic tobacco suspension cells and formulated as an injectable vaccine. Other studies on Newcastle disease virus include expression of full-size glycoproteins in transgenic potato and tobacco leaves, maize and rice seeds (reviewed in [85]). The antigens were shown to be immunogenic and protective in chickens after oral delivery.

An edible potato-based vaccine has been developed against chicken infectious bronchitis virus (IBV) [86]. Sliced tubers expressing viral S1 glycoprotein were administered in three doses over two weeks; chickens were challenged with IBV one week after the last administration. Orally immunized chickens developed a virus-specific antibody response and were protected against IBV. Another research group succeeded in vaccinating chickens against infectious bursal disease virus (IBDV) with plant-made VP2 protein. Chickens orally immunized with *Arabidopsis* crude leaf extracts or transgenic rice seeds were protected to a similar level achieved with a commercial injectable vaccine [87]. Recently, the VP2 antigen was produced transiently in *Nicotiana benthamiana* and induced neutralizing antibodies in immunized chickens [88].

#### 6.3.2 Swine

The use of maize seeds as an edible delivery vehicle against porcine Transmissible gastroenteritis coronavirus (TGEV) has been extensively studied. The envelope spike protein was used as an antigen to raise neutralizing antibodies and the efficacy of this plant-made vaccine has been presented in experiments with piglets [89]. In addition, it was found that the antigen was stable during storage in various conditions and authors were able to concentrate the antigen using milling techniques.

Enterotoxigenic *E. coli* (ETEC) causing post-weaning diarrhea in piglets has been a target for a plant-made vaccine. The major subunit protein of ETEC F4 fimbriae has been expressed in the leaves of tobacco [90], alfalfa [46] and in seeds of barley [91]. This subunit vaccine was shown to be immunogenic and partially protective after oral delivery to weaned piglets [46].

Capsid protein VP1 antigenic epitope of the of Foot-and-mouth disease virus (FMDV) was used to substitute a part of Bamboo mosaic virus coat protein, resulting in display of the epitope on virions that retained their infectivity and could be propagated in plants [92]. It was demonstrated that immunization with the extracted chimeric virions by intramuscular injection could fully protect pigs against FMDV challenge by effectively inducing humoral and cell-mediated immune responses.

#### 6.3.3 Cattle

Transgenic peanut plants expressing bovine Rinderpest virus hemaglutinin raised immune responses in cattle [93]. This oral vaccine was able to raise virus-specific antibodies which also neutralized the virus in vitro. Immunogenicity of a Tobacco mosaic virus (TMV)-based vaccine against bovine herpes virus (BHV) was studied in cattle [94]. Immunogenic glycoprotein D was produced as a by-product in TMV-inoculated tobacco plants, and the crude plant extract emulsified in oil and subsequently injected into cattle was able to raise specific humoral and cellular immune responses. Most importantly, cattle were protected against BHV to similar levels as those vaccinated with the commercial vaccine.

A plant-made subunit vaccine against Bovine viral diarrhea virus (BVDV) has been developed in Argentina. The structural protein E2 of BVDV was expressed in transgenic alfalfa as a fusion to a peptide for targeting the product to antigen-presenting cells [95]. Partially purified antigen was administered intramuscularly to calves and protected them against BVDV challenge.

### 6.4 Toxic proteins

Although the efficacy of treatments with classical antibiotics is declining due to the widespread development of antibiotic resistance among major pathogenic bacteria, there are very little alternatives for fighting infections. One novel therapeutic option could be the use of phage lysins - enzymes that target and hydrolyze bacterial cell walls, efficiently killing a large array of bacteria [96]. However, due to the toxic nature of these proteins, their production in bacteria cannot be achieved, limiting the availability for testing or manufacture. The lack of the specific, bacterial-type cell wall structures targeted by lysins makes plants an alternative platform for production of therapeutic proteins which are highly toxic to bacteria. In a promising report [97], Oey et al. showed that by targeting the genes encoding the lysozyme Cpl-1 and the amidase Pal to the tobacco plastome, functional lysins could be synthesized and accumulated at high levels in chloroplasts. Several reports described successful transplastomic as well as transient expression-mediated production of short anti-microbial peptides, analogs of secreted defensins, which can be used for control of bacterial, viral and fungal infections [98-100]. Hence, plants represent a valuable production system for anti-microbial toxic proteins, offering cost-effective solution to obtain large quantities of proteinaceous antibiotics that could be administered through feed.

## 7. Conclusions

The use of vaccines and prophylactics for the control of infectious diseases in the livestock industry will grow as antibiotics applications diminish. Plants as bioreactors comprise a valuable option for production of recombinant protein therapeutics for animal health. In recent years numerous studies demonstrated the feasibility and advantages of plant-based production platforms for various proteins with therapeutic use, including complex antibodies, subunit vaccines and immunogenic virus-like particles. Plant-made therapeutic products are currently on the cusp of widely entering biotechnology markets. Interaction and concerted actions of the plant biotechnology sector with veterinarians and regulatory authorities will facilitate development of novel approaches to sustainable, antibiotic-free livestock agriculture.

## List of abbreviations

BHV: Bovine herpes virus
BVDV: Bovine viral diarrhea virus
CT: Cholera toxin
CTB: B subunit of CT
DARPA: Defense advanced research program agency
ETEC: Enterotoxigenic *E. coli*
FcRn: Neonatal Fc receptor
FMDV: Foot-and-mouth disease virus
GALT: Gut-associated lymphoid tissue
GMP: Good Manufacturing Practice
IBV: Infectious bronchitis virus
Ig: Immunoglobulin
LT: Thermolabile enterotoxin
LTB: B subunit of LT
MALT: Mucosa-associated lymphoid tissue
PTMs: Post-translational modifications
scFv: Single-chain variable antibody fragments
TGEV: Transmissible gastroenteritis coronavirus
TMV: Tobacco mosaic virus
VLPs: Virus-like particles

## References

[1] Scallan E, Hoekstra RM, Angulo FJ, Tauxe RV, Widdowson MA, Roy SL, Jones JL, Griffin PM (2011). Foodborne illness acquired in the United States–major pathogens. Emerg Infect Dis.

[2] One Health Initiative: **One Health Initiative will unite human and veterinary medicine.** 2014. [http://www.onehealthinitiative.com/index.php] (accessed 2014/08/29).

[3] Potter A, Gerdts V, Litte-van den Hurk S (2008). Veterinary vaccines: alternatives to antibiotics?. Anim Health Res Rev.

[4] Research and Markets: **Animal/Veterinary Vaccines Market by Products, Diseases & Technology – Global Forecast to 2018.** In 2013. [http://www.researchandmarkets.com/research/gcksr4/animalveterinary] (accessed 2014/08/29).

[5] Chappuis G (1998). Neonatal immunity and immunisation in early age: lessons from veterinary medicine. Vaccine.

[6] Sangild PT (2003). Uptake of colostral immunoglobulins by the compromised newborn farm animal. Acta Vet Scand Suppl.

[7] Hurley WL, Theil PK (2011). Perspectives on immunoglobulins in colostrum and milk. Nutrients.

[8] Cervenak J, Kacskovics I (2009). The neonatal Fc receptor plays a crucial role in the metabolism of IgG in livestock animals. Vet Immunol Immunopathol.

[9] Mayer B, Kis Z, Kajan G, Frenyo LV, Hammarstrom L, Kacskovics I (2004). The neonatal Fc receptor (FcRn) is expressed in the bovine lung. Vet Immunol Immunopathol.

[10] Porter P, Noakes DE, Allen WD (1970). Secretory IgA and antibodies to Escherichia coli in porcine colostrum and milk and their significance in the alimentary tract of the young pig. Immunology.

[11] Brandtzaeg P (2013). Secretory immunity with special reference to the oral cavity. J Oral Microbiol.

[12] Snoeck V, Peters IR, Cox E (2006). The IgA system: a comparison of structure and function in different species. Vet Res.

[13] Holmgren J, Czerkinsky C (2005). Mucosal immunity and vaccines. Nat Med.

[14] Devriendt B, De Geest BG, Goddeeris BM, Cox E (2012). Crossing the barrier: Targeting epithelial receptors for enhanced oral vaccine delivery. J Control Release.

[15] Holmgren J, Svennerholm AM (2012). Vaccines against mucosal infections. Curr Opin Immunol.

[16] Cox E, Verdonck F, Vanrompay D, Goddeeris B (2006). Adjuvants modulating mucosal immune responses or directing systemic responses towards the mucosa. Vet Res.

[17] Van den Broeck W, Cox E, Goddeeris BM (1999). Receptor-dependent immune responses in pigs after oral immunization with F4 fimbriae. Infect Immun.

[18] Snoeck V, Van den Broeck W, De Colvenaer V, Verdonck F, Goddeeris B, Cox E (2008). Transcytosis of F4 fimbriae by villous and dome epithelia in F4-receptor positive pigs supports importance of receptor-dependent endocytosis in oral immunization strategies. Vet Immunol Immunopathol.

[19] Boleman SL, Boleman SJ, Morgan WW, Hale DS, Griffin DB, Savell JW, Ames RP, Smith MT, Tatum JD, Field TG, Smith GC, Gardner BA, Morgan JB, Northcutt SL, Dolezal HG, Gill DR, Ray FK (1998). National Beef Quality Audit-1995: survey of producer-related defects and carcass quality and quantity attributes. J Anim Sci.

[20] Wilson-Welder JH, Torres MP, Kipper MJ, Mallapragada SK, Wannemuehler MJ, Narasimhan B (2009). Vaccine adjuvants: current challenges and future approaches. J Pharm Sci.

[21] Lawhorn DB (2002). Atrophic rhinitis. Texas A&M AgriLife Extension Service.

[22] Garlapati S, Eng NF, Kiros T, Kindrachuk J, Mutwiri G, Hancock RE, Potter A, Babiuk LA, Gerdts V (2011). Immunization with PCEP microparticles containing pertussis toxoid, CpG ODN and a synthetic innate defense regulator peptide induce protective immunity against pertussis. Vaccine.

[23] Polewicz M, Gracia A, Garlapati S, van Kessel J, Strom S, Halperin S, Hancock RE, Potter A, Babiuk LA, Gerdts V (2013). Novel vaccine formulations against pertussis offer earlier onset of immunity and provide protection in the presence of maternal antibodies. Vaccine.

[24] Lycke N (2012). Recent progress in mucosal vaccine development: potential and limitations. Nat Rev Immunol.

[25] Zaman M, Chandrudu S, Toth I (2013). Strategies for intranasal delivery of vaccines. Drug Deliv Transl Res.

[26] Mutsch M, Zhou W, Rhodes P, Bopp M, Chen RT, Linder T, Spyr C, Steffen R (2004). **Use of the inactivated intranasal influenza vaccine and the risk of Bell**’**s palsy in Switzerland**. N Engl J Med.

[27] Johnston M, Zakharov A, Papaiconomou C, Salmasi G, Armstrong D (2004). Evidence of connections between cerebrospinal fluid and nasal lymphatic vessels in humans, non-human primates and other mammalian species. Cerebrospinal Fluid Res.

[28] Langridge WH (2000). Edible vaccines. Sci Am.

[29] Rybicki EP (2010). Plant-made vaccines for humans and animals. Plant Biotechnol J.

[30] Komarova TV, Baschieri S, Donini M, Marusic C, Benvenuto E, Dorokhov YL (2010). Transient expression systems for plant-derived biopharmaceuticals. Expert Rev Vaccines.

[31] Desai PN, Shrivastava N, Padh H (2010). Production of heterologous proteins in plants: strategies for optimal expression. Biotechnol Adv.

[32] Sharma AK, Sharma MK (2009). Plants as bioreactors: Recent developments and emerging opportunities. Biotechnol Adv.

[33] Bock R, Warzecha H (2010). Solar-powered factories for new vaccines and antibiotics. Trends Biotechnol.

[34] Tregoning JS, Nixon P, Kuroda H, Svab Z, Clare S, Bowe F, Fairweather N, Ytterberg J, van Wijk KJ, Dougan G, Maliga P (2003). Expression of tetanus toxin Fragment C in tobacco chloroplasts. Nucleic Acids Res.

[35] D’Aoust M, Couture MM, Charland N, Trépanier S, Landry N, Ors F, Vézina L (2010). The production of hemagglutinin-based virus-like particles in plants: a rapid, efficient and safe response to pandemic influenza. Plant Biotechnol J.

[36] Gleba Y, Klimyuk V, Marillonnet S (2007). Viral vectors for the expression of proteins in plants. Curr Opin Biotechnol.

[37] Medicago Inc: 2014. [http://www.medicago.com/English/Home/default.aspx] (accessed 2014/08/29).

[38] Kentucky Bioprocessing LLC: 2014. [http://www.kbpllc.com/] (accessed 2014/08/29).

[39] Werner S, Breus O, Symonenko Y, Marillonnet S, Gleba Y (2011). High-level recombinant protein expression in transgenic plants by using a double-inducible viral vector. Proc Natl Acad Sci U S A.

[40] Xu J, Ge X, Dolan MC (2011). Towards high-yield production of pharmaceutical proteins with plant cell suspension cultures. Biotechnol Adv.

[41] Walsh G (2010). Post-translational modifications of protein biopharmaceuticals. Drug Discov Today.

[42] Aviezer D, Brill-Almon E, Shaaltiel Y, Hashmueli S, Bartfeld D, Mizrachi S, Liberman Y, Freeman A, Zimran A, Galun E (2009). A plant-derived recombinant human glucocerebrosidase enzyme - a preclinical and phase I investigation. PLoS One.

[43] Peters J, Stoger E (2011). Transgenic crops for the production of recombinant vaccines and anti-microbial antibodies. Hum Vaccin.

[44] Stoger E, Ma JK, Fischer R, Christou P (2005). Sowing the seeds of success: pharmaceutical proteins from plants. Curr Opin Biotechnol.

[45] Lakshmi PS, Verma D, Yang X, Lloyd B, Daniell H (2013). Low cost tuberculosis vaccine antigens in capsules: expression in chloroplasts, bio-encapsulation, stability and functional evaluation in vitro. PLoS One.

[46] Joensuu JJ, Verdonck F, Ehrstrom A, Peltola M, Siljander-Rasi H, Nuutila AM, Oksman-Caldentey KM, Teeri TH, Cox E, Goddeeris BM, Niklander-Teeri V (2006). F4 (K88) fimbrial adhesin FaeG expressed in alfalfa reduces F4+ enterotoxigenic *Escherichia coli* excretion in weaned piglets. Vaccine.

[47] Wilken LR, Nikolov ZL (2012). Recovery and purification of plant-made recombinant proteins. Biotechnol Adv.

[48] Jacob SS, Cherian S, Sumithra TG, Raina OK, Sankar M (2013). Edible vaccines against veterinary parasitic diseases–current status and future prospects. Vaccine.

[49] Streatfield SJ (2005). Plant-based vaccines for animal health. Rev Sci Tech.

[50] Phan HT, Hause B, Hause G, Arcalis E, Stoger E, Maresch D, Altmann F, Joensuu J, Conrad U (2014). Influence of elastin-like polypeptide and hydrophobin on recombinant hemagglutinin accumulations in transgenic tobacco plants. PLoS One.

[51] Gomord V, Fitchette AC, Menu-Bouaouiche L, Saint-Jore-Dupas C, Plasson C, Michaud D, Faye L (2010). Plant-specific glycosylation patterns in the context of therapeutic protein production. Plant Biotechnol J.

[52] Hofbauer A, Stoger E (2013). Subcellular accumulation and modification of pharmaceutical proteins in different plant tissues. Curr Pharm Des.

[53] Bosch D, Castilho A, Loos A, Schots A, Steinkellner H (2013). N-glycosylation of plant-produced recombinant proteins. Curr Pharm Des.

[54] Shaaltiel Y, Bartfeld D, Hashmueli S, Baum G, Brill-Almon E, Galili G, Dym O, Boldin-Adamsky SA, Silman I, Sussman JL, Futerman AH, Aviezer D (2007). **Production of glucocerebrosidase with terminal mannose glycans for enzyme replacement therapy of Gaucher**’**s disease using a plant cell system**. Plant Biotechnol J.

[55] Castilho A, Strasser R, Stadlmann J, Grass J, Jez J, Gattinger P, Kunert R, Quendler H, Pabst M, Leonard R, Altmann F, Steinkellner H (2010). In planta protein sialylation through overexpression of the respective mammalian pathway. J Biol Chem.

[56] Gasdaska JR, Sherwood S, Regan JT, Dickey LF (2012). An afucosylated anti-CD20 monoclonal antibody with greater antibody-dependent cellular cytotoxicity and B-cell depletion and lower complement-dependent cytotoxicity than rituximab. Mol Immunol.

[57] Yang Z, Drew DP, Jorgensen B, Mandel U, Bach SS, Ulvskov P, Levery SB, Bennett EP, Clausen H, Petersen BL (2012). Engineering mammalian mucin-type O-glycosylation in plants. J Biol Chem.

[58] Takaiwa F (2013). Update on the use of transgenic rice seeds in oral immunotherapy. Immunotherapy.

[59] Khan I, Twyman RM, Arcalis E, Stoger E (2012). Using storage organelles for the accumulation and encapsulation of recombinant proteins. Biotechol J.

[60] Zimmermann J, Saalbach I, Jahn D, Giersberg M, Haehnel S, Wedel J, Macek J, Zoufal K, Glunder G, Falkenburg D, Kipriyanov S (2009). Antibody expressing pea seeds as fodder for prevention of gastrointestinal parasitic infections in chickens. BMC Biotechnol.

[61] Nochi T, Takagi H, Yuki Y, Yang L, Masumura T, Mejima M, Nakanishi U, Matsumura A, Uozumi A, Hiroi T, Morita S, Tanaka K, Takaiwa F, Kiyono H (2007). Rice-based mucosal vaccine as a global strategy for cold-chain- and needle-free vaccination. Proc Natl Acad Sci U S A.

[62] Woodleif WG, Chaplin JF, Cambell CR, Dejong DW (1981). Effect of variety and harvest treatments on protein yield of close grown tobacco. Tobacco Sci.

[63] Pogue GP, Vojdani F, Palmer KE, Hiatt E, Hume S, Phelps J, Long L, Bohorova N, Kim D, Pauly M, Velasco J, Whaley K, Zeitlin L, Garger SJ, White E, Bai Y, Haydon H, Bratcher B (2010). Production of pharmaceutical-grade recombinant aprotinin and a monoclonal antibody product using plant-based transient expression systems. Plant Biotechnol J.

[64] Cummings JF, Guerrero ML, Moon JE, Waterman P, Nielsen RK, Jefferson S, Gross FL, Hancock K, Katz JM, Yusibov V, Fraunhofer USACfMBSG (2014). Safety and immunogenicity of a plant-produced recombinant monomer hemagglutinin-based influenza vaccine derived from influenza A (H1N1)pdm09 virus: a Phase 1 dose-escalation study in healthy adults. Vaccine.

[65] ICON Genetics GmbH: 2014. [http://www.icongenetics.com/html/home.htm] (accessed 2014/08/29).

[66] Melnik S, Stoger E (2013). Green factories for biopharmaceuticals. Curr Med Chem.

[67] Qiu X, Wong G, Audet J, Bello A, Fernando L, Alimonti JB, Fausther-Bovendo H, Wei H, Aviles J, Hiatt E, Johnson A, Morton J, Swope K, Bohorov O, Bohorova N, Goodman C, Kim D, Pauly MH, Velasco J, Pettitt J, Olinger GG, Whaley K, Xu B, Strong JE, Zeitlin L, Kobinger GP (2014). Reversion of advanced Ebola virus disease in nonhuman primates with ZMapp. Nature.

[68] Lentz EM, Garaicoechea L, Alfano EF, Parreno V, Wigdorovitz A, Bravo-Almonacid FF (2012). Translational fusion and redirection to thylakoid lumen as strategies to improve the accumulation of a camelid antibody fragment in transplastomic tobacco. Planta.

[69] Rodriguez M, Ramirez NI, Ayala M, Freyre F, Perez L, Triguero A, Mateo C, Selman-Housein G, Gavilondo JV, Pujol M (2005). Transient expression in tobacco leaves of an aglycosylated recombinant antibody against the epidermal growth factor receptor. Biotechnol Bioeng.

[70] Zeitlin L, Bohorov O, Bohorova N, Hiatt A, do Kim H, Pauly MH, Velasco J, Whaley KJ, Barnard DL, Bates JT, Crowe JE, Piedra PA, Gilbert BE (2013). Prophylactic and therapeutic testing of Nicotiana-derived RSV-neutralizing human monoclonal antibodies in the cotton rat model. MAbs.

[71] Grohs BM, Niu Y, Veldhuis LJ, Trabelsi S, Garabagi F, Hassell JA, McLean MD, Hall JC (2010). Plant-produced trastuzumab inhibits the growth of HER2 positive cancer cells. J Agric Food Chem.

[72] Virdi V, Coddens A, De Buck S, Millet S, Goddeeris BM, Cox E, De Greve H, Depicker A (2013). Orally fed seeds producing designer IgAs protect weaned piglets against enterotoxigenic *Escherichia coli* infection. Proc Natl Acad Sci U S A.

[73] Ma JK, Hiatt A, Hein M, Vine ND, Wang F, Stabila P, van Dolleweerd C, Mostov K, Lehner T (1995). Generation and assembly of secretory antibodies in plants. Science.

[74] Corthesy B (1997). Recombinant secretory IgA for immune intervention against mucosal pathogens. Biochem Soc Trans.

[75] Zeltins A (2013). Construction and characterization of virus-like particles: a review. Mol Biotechnol.

[76] Spohn G, Bachmann MF (2008). Exploiting viral properties for the rational design of modern vaccines. Expert Rev Vaccines.

[77] Mason HS, Lam DM, Arntzen CJ (1992). Expression of hepatitis B surface antigen in transgenic plants. Proc Natl Acad Sci U S A.

[78] Greco R, Michel M, Guetard D, Cervantes-Gonzalez M, Pelucchi N, Wain-Hobson S, Sala F, Sala M (2007). Production of recombinant HIV-1/HBV virus-like particles in Nicotiana tabacum and Arabidopsis thaliana plants for a bivalent plant-based vaccine. Vaccine.

[79] Thuenemann EC, Meyers AE, Verwey J, Rybicki EP, Lomonossoff GP (2013). A method for rapid production of heteromultimeric protein complexes in plants: assembly of protective bluetongue virus-like particles. Plant Biotechnol J.

[80] Santi L, Batchelor L, Huang Z, Hjelm B, Kilbourne J, Arntzen CJ, Chen Q, Mason HS (2008). An efficient plant viral expression system generating orally immunogenic Norwalk virus-like particles. Vaccine.

[81] Lai H, He J, Engle M, Diamond MS, Chen Q (2012). Robust production of virus-like particles and monoclonal antibodies with geminiviral replicon vectors in lettuce. Plant Biotechnol J.

[82] Vézina L-P, D’Aoust M-A, Landry N, Couture MMJ, Charland N, Ors F, Barbeau B, Sheldon AJ: **Plants as an innovative and accelerated vaccine-manufacturing solution.** *Bio Pharm Int* 2011, Santa Monica, CA: Advanstar Communications Inc. S27–S30.

[83] Scotti N, Rybicki EP, Buonaguro FM, Buonaguro L (2014). Plant-produced virus-like particle vaccines. Virus-like Particles in Vaccine Development.

[84] Vermij P (2006). USDA approves the first plant-based vaccine. Nat Biotechnol.

[85] Joensuu JJ, Niklander-Teeri V, Brandle JE (2008). Transgenic plants for animal health: Plant-made vaccine antigens for animal infectious disease control. Phytochem Rev.

[86] Zhou JY, Cheng LQ, Zheng XJ, Wu JX, Shang SB, Wang JY, Chen JG (2004). Generation of the transgenic potato expressing full-length spike protein of infectious bronchitis virus. J Biotechnol.

[87] Wu J, Yu L, Li L, Hu J, Zhou J, Zhou X (2007). Oral immunization with transgenic rice seeds expressing VP2 protein of infectious bursal disease virus induces protective immune responses in chickens. Plant Biotechnol J.

[88] Gomez E, Lucero MS, Chimeno Zoth S, Carballeda JM, Gravisaco MJ, Berinstein A (2013). Transient expression of VP2 in Nicotiana benthamiana and its use as a plant-based vaccine against infectious bursal disease virus. Vaccine.

[89] Lamphear BJ, Jilka JM, Kesl L, Welter M, Howard JA, Streatfield SJ (2004). A corn-based delivery system for animal vaccines: an oral transmissible gastroenteritis virus vaccine boosts lactogenic immunity in swine. Vaccine.

[90] Kolotilin I, Kaldis A, Devriendt B, Joensuu J, Cox E, Menassa R (2012). Production of a subunit vaccine candidate against porcine post-weaning diarrhea in high-biomass transplastomic tobacco. PLoS One.

[91] Joensuu JJ, Kotiaho M, Teeri TH, Valmu L, Nuutila AM, Oksman-Caldentey KM, Niklander-Teeri V (2006). Glycosylated F4 (K88) fimbrial adhesin FaeG expressed in barley endosperm induces ETEC-neutralizing antibodies in mice. Transgenic Res.

[92] Yang CD, Liao JT, Lai CY, Jong MH, Liang CM, Lin YL, Lin NS, Hsu YH, Liang SM (2007). Induction of protective immunity in swine by recombinant bamboo mosaic virus expressing foot-and-mouth disease virus epitopes. BMC Biotechnol.

[93] Khandelwal A, Lakshmi Sita G, Shaila MS (2003). Oral immunization of cattle with hemagglutinin protein of rinderpest virus expressed in transgenic peanut induces specific immune responses. Vaccine.

[94] Perez Filgueira DM, Zamorano PI, Dominguez MG, Taboga O, Del Medico Zajac MP, Puntel M, Romera SA, Morris TJ, Borca MV, Sadir AM (2003). Bovine herpes virus gD protein produced in plants using a recombinant tobacco mosaic virus (TMV) vector possesses authentic antigenicity. Vaccine.

[95] Aguirreburualde MS, Gomez MC, Ostachuk A, Wolman F, Albanesi G, Pecora A, Odeon A, Ardila F, Escribano JM, Dus Santos MJ, Wigdorovitz A (2013). Efficacy of a BVDV subunit vaccine produced in alfalfa transgenic plants. Vet Immunol Immunopathol.

[96] Pastagia M, Schuch R, Fischetti VA, Huang DB (2013). Lysins: the arrival of pathogen-directed anti-infectives. J Med Microbiol.

[97] Oey M, Lohse M, Scharff LB, Kreikemeyer B, Bock R (2009). Plastid production of protein antibiotics against pneumonia via a new strategy for high-level expression of antimicrobial proteins. Proc Natl Acad Sci U S A.

[98] Zeitler B, Bernhard A, Meyer H, Sattler M, Koop HU, Lindermayr C (2013). Production of a *de-novo* designed antimicrobial peptide in *Nicotiana benthamiana*. Plant Mol Biol.

[99] Lee SB, Li B, Jin S, Daniell H (2011). Expression and characterization of antimicrobial peptides Retrocyclin-101 and Protegrin-1 in chloroplasts to control viral and bacterial infections. Plant Biotechnol J.

[100] Company N, Nadal A, La Paz JL, Martinez S, Rasche S, Schillberg S, Montesinos E, Pla M (2014). The production of recombinant cationic alpha-helical antimicrobial peptides in plant cells induces the formation of protein bodies derived from the endoplasmic reticulum. Plant Biotechnol J.

